# Massive Air Shadow in the Abdomen

**DOI:** 10.4103/1319-3767.65190

**Published:** 2010-07

**Authors:** Bilal Mirza, Lubna Ijaz, Arsalan Qureshi, Afzal Sheikh

**Affiliations:** Department of Pediatric Surgery, The Children's Hospital and The Institute of Child Health, Lahore, Pakistan

A two-month-old male infant presented in surgical emergency of our institution, with complaints of excessive crying, epigastric swelling and non bilious vomiting of one day. There was no history of such events before. On clinical examination, the infant was febrile with a temperature of 100°F, a pulse of 110/min, respiratory rate 30/min and BP within normal range. The epigastrium revealed fullness and tenderness on palpation. Bowel sounds were audible. X-ray abdomen erect was performed with AP and lateral views [Figures 1 and 2].

**Figure 1:**
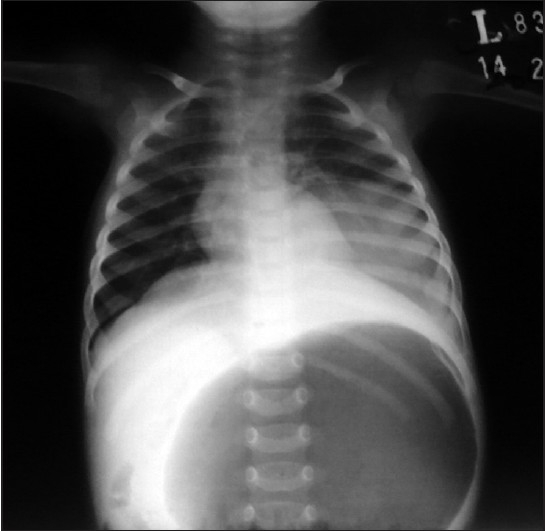
Anteroposterior view

**Figure 2:**
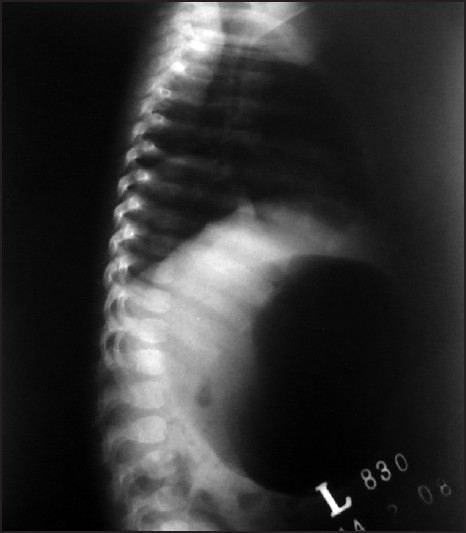
Lateral view

## QUESTIONS

1. What is the diagnosis?
2. What are the types of this condition?
3. What are the predisposing factors leading to that condition?
4. What is the management of this condition?

## ANSWERS

1. X-ray abdomen erect was showing massive gas shadow of the stomach due to volvulus around its long axis. This condition is pronounced as “acute gastric volvulus”, and defined as an abnormal degree of rotation of one part of stomach around another; resulting in partial or complete obstruction at the inlet as well as outlet of the stomach. If this condition is not managed promptly, compromise of the vascularity or gastric perforation may follow. Gastric volvulus was first reported by Berti in 1886. Gastric volvulus is a rare entity in pediatric age group. The principal symptoms include excessive crying, colicky abdominal pain, nausea, vomiting and abdominal distension more marked in the epigastrium.[1]
2. Gastric volvulus is classified as organoaxial and mesenterioaxial, according to plane around which rotation occurs. In organoaxial volvulus, the stomach rotates around its long axis causing closure of cardiac and pyloric ends of the stomach. In mesenterioaxial volvulus, rotation is on an axis from greater to lesser curvature.[1,2]
3. Gastric volvulus is uncommon because the stomach is held securely in place by the gastrophrenic ligaments, esophageal hiatus, retroperitoneal fixation of duodenum, short gastric vessels, and gastrocolic ligaments. Ligamentous laxity, pyloric obstruction leading to gastric dilatation, hiatus hernia, eventration of diaphragm, bochdalek's hernia, phrenic nerve palsy, diaphragmatic rupture, splenic mobility, splenomegaly, polysplenia, malrotation, and dislocation or hypoplasia of the left lobe of liver etc. are the predisposing factors leading to gastric volvulus.[1,3]
4. Gastric volvulus may present acutely, requiring urgent surgical intervention to prevent gastric perforation and gangrene. Simple decompression with nasogastric tube may temporarily control the emergency, but definitive treatment requires surgical intervention. During the surgery, the stomach is first decompressed and anatomy is restored. Then the predisposing factors are identified and treated. Lastly, anterior gastropexy with or without gastrostomy is performed. The recurrence is scarcely reported and gastropexy is curative.[1–4]
